# TIBLE: a web-based, freely accessible resource for small-molecule binding data for mycobacterial species

**DOI:** 10.1093/database/bax041

**Published:** 2017-06-11

**Authors:** Sony Malhotra, Grace Mugumbate, Tom L. Blundell, Alicia P. Higueruelo

**Affiliations:** a Department of Biochemistry, University of Cambridge, Tennis Court Road, Cambridge CB2 1GA, UK

## Abstract

TIBLE is a web-based resource that provides easy access to data on the minimal inhibitory concentrations for small molecules against several mycobacterial species, as well as the target binding and off-target predictions for *Mycobacterium tuberculosis*. The current version of the database holds the activity data for more than 19 000 distinct small molecules against 39 mycobacterial species, binding data for 106 *Mycobacterium tuberculosis* target proteins and predictions for their potential off-targets. The resource integrates disparate public data and methods to provide easy access to the minimum inhibitory concentration and binding data, facilitation of data sharing, and identification of small molecules and targets for development of anti-tuberculosis therapeutics.

**Database URL:** http://www-cryst.bioc.cam.ac.uk/tible/

## Introduction

The *Mycobacterium* genus includes various pathogenic and non-pathogenic species, with thick cell walls, which are rich in mycolic acid (http://www.bacterio.net/mycobacterium.html). The pathogenic mycobacterial species cause serious diseases in humans and other species, such as tuberculosis (*Mycobacterium tuberculosis*), leprosy (*Mycobacterium leprae*), ulcerated lesions (*Mycobacterium ulcerans*) and other lung infections (*Mycobacterium abscessus*). Some of these species, such as *M. tuberculosis* and *M. leprae*, are adapted to survive only as obligate pathogens and hence have reduced the size of their genomes, whereas other species, such as *M. abscessus* (opportunist pathogen which affects patients with cystic fibrosis and other lung diseases) and *M. smegmatis* (non-pathogenic), have acquired genes through horizontal gene transfer and consequently have larger genomes. Here, we collate the small-molecule binding data for all the mycobacterial species from ChEMBL database (https://www.ebi.ac.uk/chembl/) and further map the *M. tuberculosis* data to their respective protein targets.

Tuberculosis (TB), a disease caused by *M. tuberculosis*, is one of the top ten causes of deaths worldwide (1). As a result of the TB goals set by the World Health Organisation (WHO) in 2000, a reduction in the mortality rate of 47% has been reported and also the TB incidence rate has dropped by 1.5% (2). These success stories can be partly attributed to the implementation of effective data collection, sharing and monitoring. However, in 2015, TB was found responsible for the deaths of 1.4 million people, with an additional 0.4 million deaths reported for HIV patients suffering from TB. Almost 0.6 million people were infected in 2015 with either multidrug-resistant TB (MDR-TB, 480 000 new cases) or rifampicin-resistant TB (RR-TB, 100 000 new cases) (1).

The emergence of resistance to the existing antimicrobial drugs raises concerns over the success of the available treatment methods. To tackle this, we need to discover new drugs, repurpose old drugs and/or develop combination therapies. In order to do this, it would be beneficial to have easy access to the available small-molecule binding data for the target proteins of the species of interest.

Over recent years, the scientific community has witnessed an increase in the number of open-source data resources that encourage data sharing to facilitate the discovery of new anti-mycobacterial drugs. These include databases like ChEMBL (3) consisting of small bioactive molecules, binding targets and assay data, DrugBank (4) holding biochemical and pharmacological data for drugs, PubChem (5) storing activities of small molecules against biological assays, Collaborative Drug Discovery (CDD) (6) recently supporting the MM4TB (More Medicines for Tuberculosis) project (7), BindingDB (8) holding binding affinities of small molecules to protein targets and ChemBank (9) storing small molecule biological screening data. These resources are reservoirs of information on the activities of small molecules against a wide range of diseases, protein targets and organisms. Amongst these, are several databases that specifically focus on certain diseases and/or the causative bacterial pathogen. There are several resources for the *M. tuberculosis* genome such as CHOPIN, a database of three-dimensional protein structure models for the *M. tuberculosis* proteome (10), SInCRe a structural interactome computational resource for *M. tuberculosis* (11), TB Database platform, which holds curated genomic data and annotation for TB research (12), TBdreamDB specializes in TB drug resistance mutation data (13), Tuberculist provides the genome annotation for *M. tuberculosis* H37Rv strain (14). However, little attention is being given to the other mycobacterial species and there are only a few databases that provide information on other mycobacterial species such as MabsBase (15) provides annotation for *M. abscessus* ATCC19977, MycoBrowser (16) holds genomic and proteomic data for *M. tuberculosis, M. avium and M. abscessus* and a recently developed database MycoBase (17), which provides updated annotations for eleven mycobacterial species including both the pathogenic (such as *M. leprae*, *M. bovis, M. avium*) and non-pathogenic species (such as *M. smegmatis*). Some of the resources that also include other bacterial species are the TDR Target Database (18), which focuses on target identification for neglected tropical disease pathogens or the MIC database (19) which has 500 records of antibiotic compounds for about 80 microorganisms. The databases that include other pathogenic mycobacterial species are certainly useful for understanding the pathogenesis and evolutionary relationships between species. However, genome annotation of non-pathogenic species can be equally valuable, as these can be used as models to study the pathogenic species.

Extracting disease and organism specific datasets from such resources remains a challenge to most researchers in medicinal chemistry, life sciences and pharmacology, especially if they are not acquainted with the cheminformatics tools or query languages, which are needed to extract the precise and complete sets of data of interest. To contribute towards the antituberculosis research efforts, we have collated, processed and provided access to the relevant mycobacterial data i.e. Minimal Inhibitory Concentration (MIC) and small molecule binding target data.

MIC is the lowest concentration (mg/l) of an antimicrobial that prevents the visible growth of a given strain of bacteria under defined conditions, and is widely used in basic research and pharmacology to determine the most effective starting point for the evaluation of an antibiotic for a given organism and species (20). MIC data provide information that can lead to an appropriate choice of an antibiotic, thereby increasing the chances of treatment success (21). On the other hand, one of the main causes of the high attrition rates in drug development is the non-clinical toxicology (22). Proposed drug candidates often fail to be proven safe and therefore it is of paramount importance to identify possible off-targets as early as possible in the discovery pipeline.

Computational ligand-based and target-based structure-guided methods have been widely applied to predict potential targets for various bioactive compounds (23). The data obtained from these experiments have provided information on off-target binding of drugs, polypharmacology and ways of reducing adverse drug interactions. To improve visibility and access to the off-target data, some small-molecule databases like ChEMBL have incorporated information about the predicted targets for approved drugs (24).

**Figure 1.**
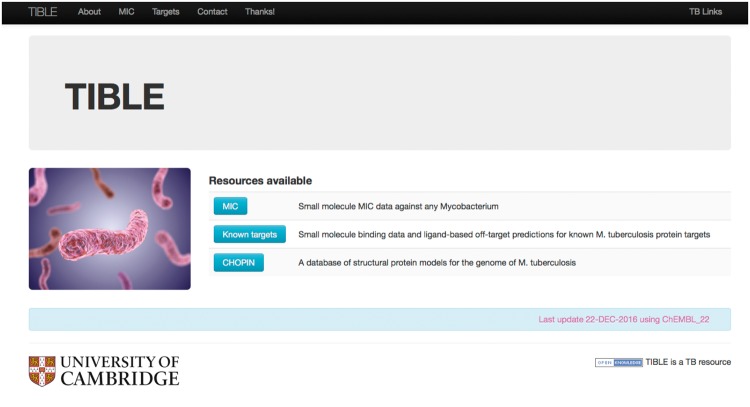
Snapshot of the TIBLE database highlighting the type of data stored for mycobacterial species.

Here, we present TIBLE (http://www-cryst.bioc.cam.ac.uk/tible/), a web-based resource that provides easy access to MIC data for small molecules against several mycobacterial species and binding data for specific protein targets for *M. tuberculosis*, including information on potential off-target sites (Figure 1). The data from TIBLE can be downloaded easily, thus encouraging data sharing, and facilitating identification of anti-TB small molecules and targets for drug discovery and development.

## Data preparation

### Sources of data

The primary source of data for TIBLE is the ChEMBL database. ChEMBL currently contains nearly 1.7 million distinct compounds, >11 000 targets, and >14 million activities that are extracted from >65 000 publications (3). ChEMBL can be accessed and downloaded as a relational database in different formats through the European Bioinformatics Institute’s website (https://www.ebi.ac.uk/chembl/). A local copy of the current version, ChEMBL_22 (in PostgreSQL), has been used to derive the TIBLE resource. Bespoke SQL queries have been developed to extract and organize mycobacterial data into the TIBLE database. MIC data points for mycobacterial species were selected from ChEMBL, by querying the compound data for assay organism as *Mycobacterium* and standard type as MIC. Further, target data for *M. tuberculosis* were retrieved from binding assays that can be mapped to a single target in the *M. tuberculosis* proteome

We have also extracted small molecule MIC and target data for *M. tuberculosis* from the CDD database for TB (6), and consolidated these data with those ChEMBL retrieved earlier in order to remove redundancy.

### Cheminformatics handling

**Figure 2.**
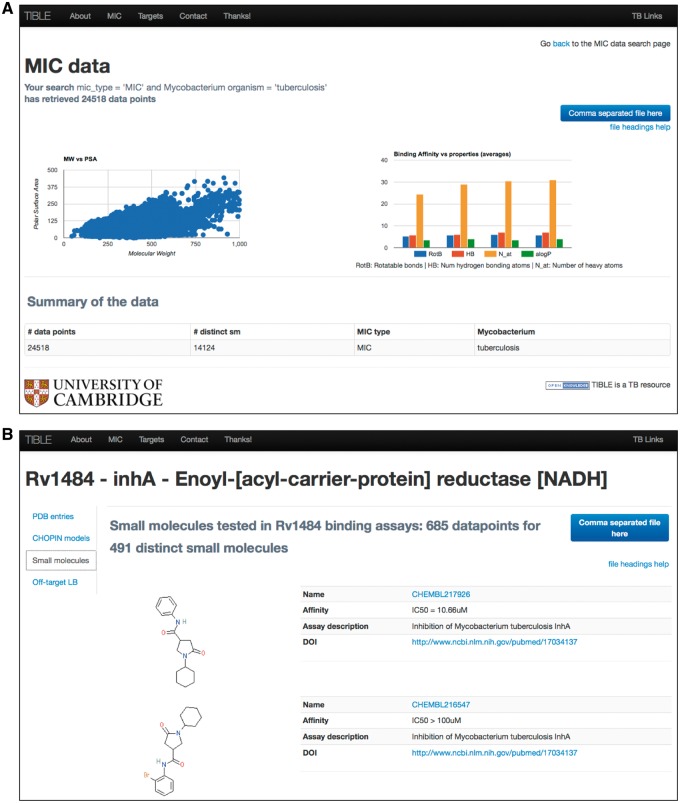
(**A**) MIC data points for *Mycobacterium tuberculosis* extracted from the ChEMBL and Collaborative Drug Discovery databases. (**B**) Small molecule binding data mapped to the target protein-Rv1484 in *Mycobacterium tuberculosis.* This page provides a link to the available protein structures in the PDB for this target protein, protein structure models in CHOPIN database and also the off-target predictions using the ligand-based methods.

RDKit (25) has been used to calculate the standard molecular properties (such as polar surface area, numbers of hydrogen bond donors and acceptors, molecular weight and rotatable bonds) and InChI (IUPAC International Chemical Identifier) for the small molecules. The main layer InChI, which holds the information about chemical formulae, atom connections and hydrogen atoms, has been used to identify the compound duplicates between the data points obtained from ChEMBL and CDD. Molecular properties of the small molecules stored in TIBLE were mapped to their MIC data to highlight regions of chemical space that are most likely to pass across the cell wall of the *Mycobacterium* (see Figure 2A).

### Off-target prediction

The computational prediction of the off-site activity is based on the similarity principle (23). A single therapeutic candidate shows activity for its intended molecular target, but is usually seen to have activity against the similar targets as well. This similarity can be described using both target-based and ligand-based approaches. In the structure-based off-target predictions, molecular targets are linked by the similarity of their pockets (or binding sites), based on the observation that similar pockets tend to bind similar molecules (23). In the case of ligand-based predictions, molecular targets are linked by the similarity of their ligands, as similar ligands tend to bind to the similar pockets. The two approaches complement each other (23) and give a more comprehensive list of possible off-targets.

We have focused on the ligand-based predictions using three different methods to predict the possible molecular off-targets for the small molecules in TIBLE that bind to *M. tuberculosis* proteins. These approaches use different algorithms and rationales but the query data are solely small molecules (or ligands). The output of the query is a ranked list of protein targets that are likely to bind the query ligands. The term ‘target fishing’ (26) has been used to describe these methodologies.

The first approach, SEA (Similarity Ensemble Approach) (27, 28), considers all ligands binding to a particular target and performs a series of pairwise comparisons against a database of targets and their ligands. This approach has been validated experimentally and has the advantage that predictions do not correlate with an absolute chemical similarity of the ligands. The second method used is PharmMapper (29) that uses a database of pharmacophores extracted from known drug targets in the PDB (30). Clustered representative ligands from an *M. tuberculosis* target are aligned with these pharmacophores and the most likely targets are identified. The advantage of this method is that it provides a structural interpretation of the off-target binding. The third method, PASS (31), is a quick assessment of toxic and undesirable side effects, based on chemical similarity against a database of small molecules with known biological activity.

The results of these predictions are accessible in TIBLE through the target pages. For the *M. tuberculosis* proteins that have small molecule binding data, we have a list of possible off-targets for that protein. For example, CHEMBL259138 is one of the cluster representatives for *inhA* (Rv1484). The top ranked predicted off-target for this ligand is the cellular retinoic acid-binding protein 2, which is in agreement with previous findings by Kinnings *et al.* (32).

## Data content

### MIC

The current version of the TIBLE database contains 44 302 MIC data points, for 19 469 unique small molecules active against 39 nontuberculous and tuberculous mycobacterial species. Apart from *M. tuberculosis*, the data points are included for other mycobacterial species such as *M. abscessus*, *M. leprae*, *M. kansasii*, *M. intracellulare* and *M. smegmatis.* All data in TIBLE are also downloadable as flat text files.

MIC data can be searched based on the mycobacterial species of interest and/or the specific MIC type: standard MIC (MIC), MIC100, MIC90, MIC99, MIC97, MIC95, MIC80, MIC50 for data that showed 100%, 90%, 99%,97%, 95%, 80% and 50% visual inhibition of the growth of the *Mycobacterium*, respectively. MIC data are also presented in the context of the molecular properties of the small molecules. Figure 2A exemplifies this by focusing on the 24 518 MIC data points specific for *M. tuberculosis*. By plotting polar surface area vs. the molecular weight, it is evident that the increase in the size of the molecule is also accompanied by a general increase in the polar surface area. This observation is in accordance with the previously reported observation that antibacterials have high molecular weight, lower logP and high polar surface area (33). Intriguingly, there is no correlation of the lipophilicity of the *M. tuberculosis* inhibitors with their binding affinities (a common trend in synthetic-molecule binding data) (34). The average number of hydrogen bonds and rotatable bonds increase steadily with the binding affinity (Figure 2A). Further in-depth analyses are required to understand these relationships. For example, it has been shown that highly polar compounds tend to bind to large binding pockets, for example in riboproteins (33), and this could explain the profile of antibacterials if many target this protein class. In addition, natural product-like molecules also exhibit more polar interactions (35) and this could contribute to the overall behavior of antituberculosis compounds if they are predominantly from natural sources.

### Target data

Target activity data for 106 *M. tuberculosis* protein targets are included in TIBLE. The target search page allows *M. tuberculosis* proteins to be found by RvID, UniProt ID or common name. There are 3923 binding data points from 2707 unique small molecules.

Each target page has four tabs: PDB entries corresponding to the respective target id, protein-structure models from the CHOPIN database, small molecules targeting this protein and the off-target predictions. The default tab is linked to the small-molecule activity data, which lists binding data points with links to original data, two-dimensional representation of small molecule(s) and a brief description of the assay. The user can navigate to the other tabs in the target page to find experimental 3D structures of the protein target if they are in the PDB (30), or predicted models from the CHOPIN database (10). Finally the off-target predictions tab will present the protein off-target predictions based on the small-molecule binding to that particular target with the methods already described: SEA (27, 28), PharmMapper (29) and PASS (31). All these data can be accessed and downloaded from the website pages.

The *M. tuberculosis* protein, Rv1484 (*inhA*), which is involved in fatty acid biosynthesis pathway and is a well-studied drug (isoniazid) target for TB (36), has the greatest number of small molecule binding data points, with activity recorded for nearly 500 distinct small molecules (Figure 2B).

## Web-resource

The TIBLE web site is a lightweight Python web framework. All data extracted from ChEMBL and CCD are stored in a PostgreSQL database (http://www.postgresql.org). SQLAlchemy (http://www.sqlalchemy.org) generates python objects from the database tables and Flask (http://flask.pocoo.org) uses these objects to create dynamic pages. We have used Bootstrap (http://twitter.github.com/bootstrap) to give the CSS (Cascading Style Sheets) framework and JavaScript functionality to create slick pages.

## Conclusion

We present TIBLE, an example of integration of disparate public data and methods, in order to provide an easy open-access resource for small-molecule binding data for several mycobacterial species. It provides data on small-molecules for their anti-mycobacterial properties, chemical properties and targets. We believe that TIBLE adds to the existing information resources for mycobacterial species in a way that will assist drug development for tackling *M. tuberculosis* infection and managing drug resistance.

## Funding

Wellcome Trust Seeding Drug Discovery (101134/Z/13/Z to A.P.H., T.L.B.); MRC Newton Award (RG78439 to S.M. and T.L.B.); Programme Grant (093167/Z/10/Z to T.L.B.); Cystic Fibrosis Trust Grant (RG 70975); Wellcome Trust Investigator Award (200814/Z/16/Z to T.L.B.). 

*Conflict of interest*. None declared.
